# *catena*-Poly[diacridinium [zinc(II)-di-μ-pyrazine-2,3-dicarboxyl­ato-κ^3^ *N* ^1^,*O* ^2^:*O* ^3^;*O* ^3^:*N* ^1^,*O* ^2^]]

**DOI:** 10.1107/S1600536810025195

**Published:** 2010-07-03

**Authors:** Hossein Eshtiagh-Hosseini, Hossein Aghabozorg, Masoud Mirzaei

**Affiliations:** a Department of Chemistry, School of Sciences, Ferdowsi University of Mashhad, Mashhad, Iran; b Faculty of Chemistry, Islamic Azad University, North Tehran Branch, Tehran, Iran

## Abstract

The crystal structure of the title compound, {(C_13_H_10_N)_2_[Zn(C_6_H_2_N_2_O_4_)_2_]}_*n*_, consists of polymeric Zn complex anions and discrete acridinium cations. The Zn cation, located on an inversion center, is *N*,*O*-chelated by two pyrazine-2,3-dicarboxyl­ate (pyzdc) anions in the basal plane, and is further coordinated by two carboxyl­ate O atoms from adjacent pyzdc anions in the axial directions with a longer Zn—O bond distance, forming a distorted ZnN_2_O_4_ coordination geometry. The pyzdc anions bridge the Zn cations, forming polymeric chains running along the crystallographic *b* axis. The acridinium cations are linked to the complex chains *via* N—H⋯O and C—H⋯O hydrogen bonding. Significant π–π stacking between parallel acridinium ring systems is observed in the crystal structure, face-to-face distances being 3.311 (3) and 3.267 (4) Å.

## Related literature

For the structure of a related Co(II) complex with pyzdc ligands, see: Aghabozorg *et al.* (2010*b* ▶). For the proton transfer of the carboxyl group, see: Aghabozorg *et al.* (2010*a* ▶). 
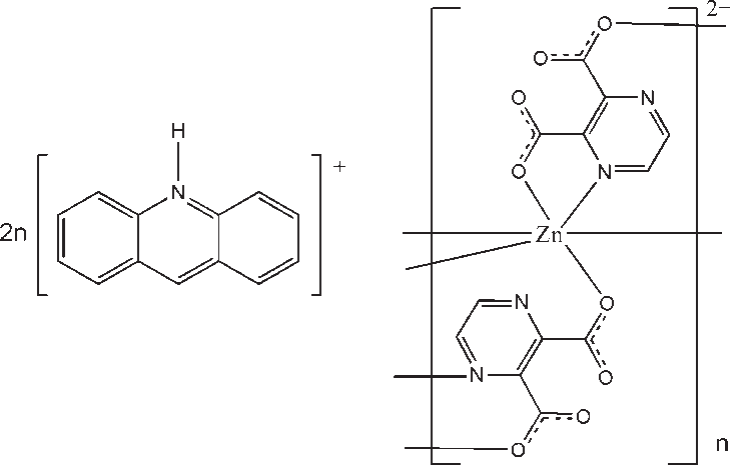


## Experimental

### 

#### Crystal data

(C_13_H_10_N)_2_[Zn(C_6_H_2_N_2_O_4_)_2_]
*M* *_r_* = 758.00
Monoclinic, 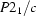

*a* = 13.2256 (12) Å
*b* = 6.8141 (6) Å
*c* = 17.9889 (16) Å
β = 111.013 (2)°
*V* = 1513.4 (2) Å^3^
*Z* = 2
Mo *K*α radiation
μ = 0.88 mm^−1^
*T* = 120 K
0.27 × 0.15 × 0.13 mm

#### Data collection

Bruker SMART 1000 CCD area-detector diffractometer
Absorption correction: multi-scan (*SADABS*; Sheldrick 1998 ▶) *T*_min_ = 0.845, *T*_max_ = 0.891
15968 measured reflections
2720 independent reflections
2292 reflections with *I* > 2σ(*I*)
*R*_int_ = 0.032

#### Refinement

*R*[*F* ^2^ > 2σ(*F* ^2^)] = 0.042
*wR*(*F* ^2^) = 0.115
*S* = 1.14
2720 reflections
244 parameters
1 restraint
H atoms treated by a mixture of independent and constrained refinement
Δρ_max_ = 0.76 e Å^−3^
Δρ_min_ = −0.47 e Å^−3^

### 

Data collection: *SMART* (Bruker, 1998 ▶); cell refinement: *SAINT-Plus* (Bruker, 1998 ▶); data reduction: *SAINT-Plus*; program(s) used to solve structure: *SHELXTL* (Sheldrick, 2008 ▶); program(s) used to refine structure: *SHELXTL*; molecular graphics: *SHELXTL*; software used to prepare material for publication: *SHELXTL*.

## Supplementary Material

Crystal structure: contains datablocks I, global. DOI: 10.1107/S1600536810025195/xu2784sup1.cif

Structure factors: contains datablocks I. DOI: 10.1107/S1600536810025195/xu2784Isup2.hkl

Additional supplementary materials:  crystallographic information; 3D view; checkCIF report

## Figures and Tables

**Table 1:** Selected bond lengths (Å)

Zn1—O1	2.0326 (17)
Zn1—N1	2.093 (2)
Zn1—O4^i^	2.2435 (17)

Symmetry code: (i) 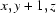.

**Table 2:** Hydrogen-bond geometry (Å, °)

*D*—H⋯*A*	*D*—H	H⋯*A*	*D*⋯*A*	*D*—H⋯*A*
N3—H3*N*⋯O3	0.86 (2)	1.78 (2)	2.634 (3)	172 (3)
C13—H13⋯O2^ii^	0.93	2.38	3.211 (4)	149
C16—H16⋯O3^iii^	0.93	2.49	3.377 (3)	159

Symmetry codes: (ii) 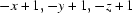; (iii) 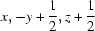.

**Fig. 1.**
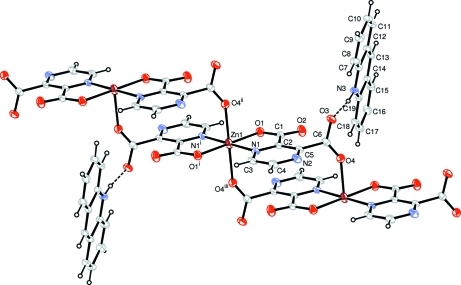
A part of the title polymeric compound, thermal ellipsoids are shown at the 50% probability level. Symmetry code: (i): 2-x, 2-y, 1-z; (ii): x, 1+y, z; (iii): 2-x, 1-y, 1-z.

## supplementary crystallographic information

### Comment

H_2_pyzdc has proved to be well suited for the construction of multidimensional frameworks due to the presence of two adjacent carboxylate groups (O donor atoms) as substituents on the N-heterocyclic pyrazine ring (N donor atoms). In this paper, we report the hydrothermal synthesis, crystal and molecular structure of a pyrazinecarboxylate-based Zn atom supramolecular coordination compound as a novel inorganic polymer, for the first time. The hydrothermal reaction between H_2_pyzdc, acr, and zinc nitrate tetra-hydrate, resulted in the formation of {(C_13_H_10_N)_2_[Zn(C_6_H_2_N_2_O_4_)_2_]}_n_. This inorganic polymeric compound consists of an anionic complex, [Zn(pyzdc)_2_]^2–^, counter-ions, (acrH)^+^ molecules. In the title inorganic polymeric compound, two COOH protons have been transferred to non-coordinated pyridine rings of acr moieties. The central Zn1 atom is six-coordinated by N1 and O1 atoms in the equatorial plane from two (pyzdc)^2–^ ligands and by two O4 atoms in the axial positions (Fig. 1). The coordination environment around the Zn atom may be considered as slightly distorted octahedral. The anionic complex lies on a crystallographic center of symmetry. The mean Zn–N and Zn–O bond lengths are 2.093 (2) and 2.138 (17) Å, respectively. In the structure of the title inorganic polymeric compound, (acrH)^+^ cations and [Zn(pyzdc)_2_]^2-^ anions are linked together by classical N3–H3B···O3 and non-classical C13–H13···O2 and C16–H16···O3 hydrogen bonds. In the crystal structure of the title polymeric compound, the spaces between [Zn(pyzdc)_2_]^2-^ fragments are filled with layers of (acrH)^+^ cations. Indeed, the arrangement of anionic layers to each other resulted in the making of suitable spaces for entering cationic parts. As a essential factor extensive π–π stacking interactions between parallel aromatic rings of the acridinium ions,(acrH)^+^, with face-to-face distances of 3.311 (3) and 3.267 (4) Å, caused to further stabilization of crystalline network.

It should be noted that most of the molecular structures consisting up dicarboxylate ligands incorporate water molecules of hydration which may lead to formation kind of (H_2_O)_n_ clusters (Review article by Aghabozorg *et al.* 2010*a*). The used reaction conditions such as hydrothermal synthesis *versus* just normal synthesis in aqueous conditions play basic roles in this regard. Additionally, if water molecules are present, it may prevent polymerization because it will coordinate to the metal center and so, used dicarboxylate ligand can not play chelate role for connecting metal centers to each other. For example, herein, we have obtained an inorganic polymer because of applying hydrothermal condition. But, recently published work of our research group (Aghabozorg *et al.* 2010*b*) show that the reaction of cobalt(II) nitrate hexa-hydrate, acr, and H_2_pyzdc in aqueous solution and routine condition resulted in the formation of (acrH)_2_[Co(pyzdc)_2_(H_2_O)_2_]. 6H_2_O crystals as monomeric structure.

### Experimental

A mixture of H_2_pyzdc (0.83 mmol, 140 mg), acridine (1.67 mmol, 300 mg), and Zn(NO_3_)_2_.4H_2_O (0.27 mmol, 80 mg) in distilled water (12 ml) was placed in a Teflon-lined stainless steel vessel, heated to 423 K for 4 days, and then cooled to room temperature over 12 h. Red block crystals were obtained after five months by slow evaporation of solvent with a yield of approximate 55% based on Zn.

### Refinement

N-bonded H atom was located in a difference Fourier map and refined with a distance restraint. Other H atoms were placed in calculated positions and refined in a riding mode. U_iso_(H) = 1.2 U_eq_(C,N).

### Crystal data

**Table:**

(C_13_H_10_N)_2_[Zn(C_6_H_2_N_2_O_4_)_2_]	*F*(000) = 776
*M**_r_* = 758.00	*D*_x_ = 1.663 Mg m^−^^3^
Monoclinic, *P*2_1_/*c*	Mo *K*α radiation, λ = 0.71073 Å
Hall symbol: -P 2ybc	Cell parameters from 600 reflections
*a* = 13.2256 (12) Å	θ = 2.0–24.0°
*b* = 6.8141 (6) Å	µ = 0.88 mm^−^^1^
*c* = 17.9889 (16) Å	*T* = 120 K
β = 111.013 (2)°	Prism, red
*V* = 1513.4 (2) Å^3^	0.27 × 0.15 × 0.13 mm
*Z* = 2	

### Data collection

**Table:**

Bruker SMART 1000 CCD area-detector diffractometer	2720 independent reflections
Radiation source: fine-focus sealed tube	2292 reflections with *I* > 2σ(*I*)
graphite	*R*_int_ = 0.032
ω scans	θ_max_ = 25.2°, θ_min_ = 1.7°
Absorption correction: multi-scan (*SADABS*; Sheldrick 1998)	*h* = −15→15
*T*_min_ = 0.845, *T*_max_ = 0.891	*k* = −8→8
15968 measured reflections	*l* = −21→21

### Refinement

**Table:**

Refinement on *F*^2^	Primary atom site location: structure-invariant direct methods
Least-squares matrix: full	Secondary atom site location: difference Fourier map
*R*[*F*^2^ > 2σ(*F*^2^)] = 0.042	Hydrogen site location: inferred from neighbouring sites
*wR*(*F*^2^) = 0.115	H atoms treated by a mixture of independent and constrained refinement
*S* = 1.14	*w* = 1/[σ^2^(*F*_o_^2^) + (0.0644*P*)^2^ + 1.454*P*] where *P* = (*F*_o_^2^ + 2*F*_c_^2^)/3
2720 reflections	(Δ/σ)_max_ = 0.001
244 parameters	Δρ_max_ = 0.76 e Å^−^^3^
1 restraint	Δρ_min_ = −0.47 e Å^−^^3^

### Special details

**Table:**

Geometry. All esds (except the esd in the dihedral angle between two l.s. planes) are estimated using the full covariance matrix. The cell esds are taken into account individually in the estimation of esds in distances, angles and torsion angles; correlations between esds in cell parameters are only used when they are defined by crystal symmetry. An approximate (isotropic) treatment of cell esds is used for estimating esds involving l.s. planes.
Refinement. Refinement of F^2^ against ALL reflections. The weighted R-factor wR and goodness of fit S are based on F^2^, conventional R-factors R are based on F, with F set to zero for negative F^2^. The threshold expression of F^2^ > 2sigma(F^2^) is used only for calculating R-factors(gt) etc. and is not relevant to the choice of reflections for refinement. R-factors based on F^2^ are statistically about twice as large as those based on F, and R- factors based on ALL data will be even larger.

### Fractional atomic coordinates and isotropic or equivalent isotropic displacement parameters (Å^2^)

**Table:**

	*x*	*y*	*z*	*U*_iso_*/*U*_eq_	
Zn1	1.0000	1.0000	0.5000	0.02299 (17)	
N1	0.97579 (16)	0.7741 (3)	0.41650 (12)	0.0181 (4)	
N2	0.92414 (17)	0.4493 (3)	0.31697 (13)	0.0235 (5)	
N3	0.56207 (16)	0.2835 (3)	0.40993 (12)	0.0188 (4)	
O1	0.90033 (14)	0.8249 (3)	0.53444 (11)	0.0225 (4)	
O2	0.81853 (16)	0.5307 (3)	0.51045 (12)	0.0275 (5)	
O3	0.71207 (14)	0.3430 (3)	0.34784 (11)	0.0231 (4)	
O4	0.84996 (14)	0.1517 (3)	0.41985 (10)	0.0219 (4)	
C1	0.87279 (19)	0.6642 (4)	0.49636 (15)	0.0183 (5)	
C2	0.91244 (18)	0.6323 (4)	0.42725 (14)	0.0166 (5)	
C3	1.0143 (2)	0.7552 (4)	0.35783 (15)	0.0213 (5)	
H3	1.0591	0.8517	0.3499	0.026*	
C4	0.9879 (2)	0.5930 (4)	0.30892 (15)	0.0234 (6)	
H4	1.0158	0.5833	0.2684	0.028*	
C5	0.88694 (19)	0.4691 (4)	0.37672 (15)	0.0174 (5)	
C6	0.8114 (2)	0.3068 (4)	0.38376 (15)	0.0197 (5)	
C7	0.4615 (2)	0.3287 (3)	0.35817 (15)	0.0173 (5)	
C8	0.4441 (2)	0.3677 (4)	0.27753 (15)	0.0216 (5)	
H8	0.5016	0.3642	0.2593	0.026*	
C9	0.3419 (2)	0.4107 (4)	0.22648 (16)	0.0233 (6)	
H9	0.3300	0.4348	0.1731	0.028*	
C10	0.2537 (2)	0.4193 (4)	0.25332 (15)	0.0233 (6)	
H10	0.1850	0.4504	0.2176	0.028*	
C11	0.2682 (2)	0.3826 (4)	0.33062 (16)	0.0229 (6)	
H11	0.2095	0.3882	0.3475	0.027*	
C12	0.3732 (2)	0.3354 (4)	0.38588 (15)	0.0187 (5)	
C13	0.3932 (2)	0.2948 (3)	0.46547 (15)	0.0183 (5)	
H13	0.3366	0.3014	0.4846	0.022*	
C14	0.4961 (2)	0.2445 (4)	0.51706 (15)	0.0183 (5)	
C15	0.5195 (2)	0.1983 (4)	0.59890 (15)	0.0219 (6)	
H15	0.4644	0.2036	0.6196	0.026*	
C16	0.6211 (2)	0.1467 (4)	0.64685 (16)	0.0239 (6)	
H16	0.6358	0.1182	0.7003	0.029*	
C17	0.7049 (2)	0.1364 (4)	0.61494 (16)	0.0237 (6)	
H17	0.7738	0.0977	0.6480	0.028*	
C18	0.6877 (2)	0.1814 (4)	0.53774 (15)	0.0210 (5)	
H18	0.7441	0.1759	0.5184	0.025*	
C19	0.5823 (2)	0.2368 (3)	0.48739 (14)	0.0180 (5)	
H3N	0.6133 (15)	0.292 (4)	0.3912 (15)	0.022*	

### Atomic displacement parameters (Å^2^)

**Table:**

	*U*^11^	*U*^22^	*U*^33^	*U*^12^	*U*^13^	*U*^23^
Zn1	0.0244 (3)	0.0228 (3)	0.0256 (3)	−0.00764 (17)	0.0137 (2)	−0.00581 (17)
N1	0.0126 (10)	0.0220 (11)	0.0196 (10)	0.0012 (8)	0.0058 (8)	0.0009 (9)
N2	0.0187 (11)	0.0312 (12)	0.0221 (11)	−0.0029 (9)	0.0093 (9)	−0.0022 (9)
N3	0.0152 (11)	0.0215 (11)	0.0217 (11)	−0.0001 (8)	0.0090 (9)	0.0011 (9)
O1	0.0224 (9)	0.0225 (9)	0.0252 (10)	−0.0031 (7)	0.0118 (8)	−0.0029 (7)
O2	0.0303 (11)	0.0281 (10)	0.0318 (11)	−0.0089 (8)	0.0205 (9)	−0.0043 (8)
O3	0.0149 (9)	0.0282 (10)	0.0251 (10)	−0.0018 (7)	0.0058 (8)	0.0009 (8)
O4	0.0209 (9)	0.0198 (9)	0.0234 (9)	−0.0017 (7)	0.0059 (8)	−0.0002 (7)
C1	0.0142 (12)	0.0211 (13)	0.0193 (13)	0.0010 (10)	0.0058 (10)	0.0012 (10)
C2	0.0120 (11)	0.0199 (12)	0.0177 (12)	0.0017 (9)	0.0051 (10)	0.0034 (10)
C3	0.0131 (12)	0.0285 (13)	0.0226 (13)	−0.0016 (10)	0.0067 (10)	0.0028 (11)
C4	0.0181 (13)	0.0320 (15)	0.0226 (13)	0.0014 (11)	0.0102 (11)	−0.0014 (11)
C5	0.0109 (11)	0.0223 (12)	0.0177 (12)	0.0004 (9)	0.0035 (10)	0.0003 (10)
C6	0.0164 (12)	0.0248 (13)	0.0182 (12)	−0.0031 (10)	0.0065 (10)	−0.0050 (10)
C7	0.0161 (12)	0.0150 (11)	0.0207 (13)	−0.0002 (9)	0.0064 (10)	−0.0015 (9)
C8	0.0201 (13)	0.0250 (13)	0.0226 (13)	0.0016 (10)	0.0110 (11)	0.0003 (11)
C9	0.0267 (14)	0.0232 (13)	0.0186 (13)	0.0004 (11)	0.0066 (11)	−0.0013 (10)
C10	0.0156 (13)	0.0259 (13)	0.0233 (14)	0.0011 (10)	0.0009 (11)	0.0021 (11)
C11	0.0149 (12)	0.0256 (13)	0.0284 (14)	−0.0010 (10)	0.0081 (11)	0.0008 (11)
C12	0.0165 (12)	0.0188 (12)	0.0224 (13)	−0.0008 (9)	0.0088 (11)	−0.0016 (10)
C13	0.0165 (12)	0.0187 (12)	0.0231 (13)	−0.0027 (10)	0.0112 (10)	−0.0028 (10)
C14	0.0177 (12)	0.0158 (12)	0.0223 (13)	−0.0035 (9)	0.0083 (10)	−0.0022 (10)
C15	0.0233 (13)	0.0232 (13)	0.0231 (13)	−0.0040 (11)	0.0131 (11)	−0.0009 (10)
C16	0.0250 (14)	0.0257 (13)	0.0203 (13)	−0.0065 (11)	0.0073 (11)	0.0017 (10)
C17	0.0164 (13)	0.0265 (13)	0.0247 (14)	−0.0015 (10)	0.0032 (11)	0.0050 (11)
C18	0.0152 (12)	0.0232 (13)	0.0252 (14)	−0.0020 (10)	0.0080 (11)	0.0010 (10)
C19	0.0194 (13)	0.0163 (11)	0.0199 (12)	−0.0032 (10)	0.0090 (10)	0.0000 (10)

### Geometric parameters (Å, °)

**Table:**

Zn1—O1	2.0326 (17)	C7—C8	1.410 (4)
Zn1—O1^i^	2.0326 (17)	C7—C12	1.425 (3)
Zn1—N1^i^	2.093 (2)	C8—C9	1.366 (4)
Zn1—N1	2.093 (2)	C8—H8	0.9300
Zn1—O4^ii^	2.2435 (17)	C9—C10	1.414 (4)
Zn1—O4^iii^	2.2435 (17)	C9—H9	0.9300
N1—C3	1.332 (3)	C10—C11	1.357 (4)
N1—C2	1.338 (3)	C10—H10	0.9300
N2—C4	1.333 (4)	C11—C12	1.425 (4)
N2—C5	1.340 (3)	C11—H11	0.9300
N3—C7	1.358 (3)	C12—C13	1.387 (4)
N3—C19	1.359 (3)	C13—C14	1.388 (4)
N3—H3N	0.86 (2)	C13—H13	0.9300
O1—C1	1.273 (3)	C14—C19	1.422 (3)
O2—C1	1.240 (3)	C14—C15	1.427 (4)
O3—C6	1.263 (3)	C15—C16	1.357 (4)
O4—C6	1.249 (3)	C15—H15	0.9300
C1—C2	1.528 (3)	C16—C17	1.422 (4)
C2—C5	1.399 (3)	C16—H16	0.9300
C3—C4	1.377 (4)	C17—C18	1.359 (4)
C3—H3	0.9300	C17—H17	0.9300
C4—H4	0.9300	C18—C19	1.414 (4)
C5—C6	1.526 (3)	C18—H18	0.9300
			
O1—Zn1—O1^i^	180.0	O3—C6—C5	114.1 (2)
O1—Zn1—N1^i^	99.32 (7)	N3—C7—C8	120.4 (2)
O1^i^—Zn1—N1^i^	80.68 (7)	N3—C7—C12	119.5 (2)
O1—Zn1—N1	80.68 (7)	C8—C7—C12	120.1 (2)
O1^i^—Zn1—N1	99.32 (7)	C9—C8—C7	119.3 (2)
N1^i^—Zn1—N1	180.0	C9—C8—H8	120.4
O1—Zn1—O4^ii^	86.88 (7)	C7—C8—H8	120.4
O1^i^—Zn1—O4^ii^	93.12 (7)	C8—C9—C10	121.2 (2)
N1^i^—Zn1—O4^ii^	89.71 (7)	C8—C9—H9	119.4
N1—Zn1—O4^ii^	90.29 (7)	C10—C9—H9	119.4
O1—Zn1—O4^iii^	93.12 (7)	C11—C10—C9	120.8 (2)
O1^i^—Zn1—O4^iii^	86.88 (7)	C11—C10—H10	119.6
N1^i^—Zn1—O4^iii^	90.29 (7)	C9—C10—H10	119.6
N1—Zn1—O4^iii^	89.71 (7)	C10—C11—C12	120.1 (2)
O4^ii^—Zn1—O4^iii^	180.000 (1)	C10—C11—H11	120.0
C3—N1—C2	118.8 (2)	C12—C11—H11	120.0
C3—N1—Zn1	129.49 (17)	C13—C12—C11	122.9 (2)
C2—N1—Zn1	111.71 (16)	C13—C12—C7	118.5 (2)
C4—N2—C5	116.2 (2)	C11—C12—C7	118.6 (2)
C7—N3—C19	122.7 (2)	C12—C13—C14	121.2 (2)
C7—N3—H3N	115.6 (19)	C12—C13—H13	119.4
C19—N3—H3N	121.6 (19)	C14—C13—H13	119.4
C1—O1—Zn1	115.63 (15)	C13—C14—C19	119.0 (2)
C6—O4—Zn1^iv^	145.70 (16)	C13—C14—C15	122.8 (2)
O2—C1—O1	126.6 (2)	C19—C14—C15	118.2 (2)
O2—C1—C2	117.0 (2)	C16—C15—C14	120.8 (2)
O1—C1—C2	116.4 (2)	C16—C15—H15	119.6
N1—C2—C5	119.9 (2)	C14—C15—H15	119.6
N1—C2—C1	115.5 (2)	C15—C16—C17	119.6 (2)
C5—C2—C1	124.7 (2)	C15—C16—H16	120.2
N1—C3—C4	120.1 (2)	C17—C16—H16	120.2
N1—C3—H3	119.9	C18—C17—C16	122.1 (2)
C4—C3—H3	119.9	C18—C17—H17	118.9
N2—C4—C3	123.1 (2)	C16—C17—H17	118.9
N2—C4—H4	118.5	C17—C18—C19	118.7 (2)
C3—C4—H4	118.5	C17—C18—H18	120.6
N2—C5—C2	121.9 (2)	C19—C18—H18	120.6
N2—C5—C6	115.7 (2)	N3—C19—C18	120.4 (2)
C2—C5—C6	122.3 (2)	N3—C19—C14	119.1 (2)
O4—C6—O3	126.0 (2)	C18—C19—C14	120.5 (2)
O4—C6—C5	119.9 (2)		
			
O1—Zn1—N1—C3	179.3 (2)	N2—C5—C6—O4	83.9 (3)
O1^i^—Zn1—N1—C3	−0.7 (2)	C2—C5—C6—O4	−98.6 (3)
O4^ii^—Zn1—N1—C3	92.5 (2)	N2—C5—C6—O3	−93.2 (3)
O4^iii^—Zn1—N1—C3	−87.5 (2)	C2—C5—C6—O3	84.3 (3)
O1—Zn1—N1—C2	−1.26 (16)	C19—N3—C7—C8	−177.2 (2)
O1^i^—Zn1—N1—C2	178.74 (16)	C19—N3—C7—C12	2.4 (4)
O4^ii^—Zn1—N1—C2	−88.05 (16)	N3—C7—C8—C9	179.3 (2)
O4^iii^—Zn1—N1—C2	91.95 (16)	C12—C7—C8—C9	−0.4 (4)
N1^i^—Zn1—O1—C1	−177.15 (17)	C7—C8—C9—C10	0.8 (4)
N1—Zn1—O1—C1	2.85 (17)	C8—C9—C10—C11	−0.8 (4)
O4^ii^—Zn1—O1—C1	93.66 (17)	C9—C10—C11—C12	0.2 (4)
O4^iii^—Zn1—O1—C1	−86.34 (17)	C10—C11—C12—C13	−179.4 (2)
Zn1—O1—C1—O2	175.2 (2)	C10—C11—C12—C7	0.2 (4)
Zn1—O1—C1—C2	−3.8 (3)	N3—C7—C12—C13	−0.1 (3)
C3—N1—C2—C5	−0.7 (3)	C8—C7—C12—C13	179.5 (2)
Zn1—N1—C2—C5	179.80 (17)	N3—C7—C12—C11	−179.8 (2)
C3—N1—C2—C1	179.3 (2)	C8—C7—C12—C11	−0.2 (3)
Zn1—N1—C2—C1	−0.2 (2)	C11—C12—C13—C14	178.3 (2)
O2—C1—C2—N1	−176.4 (2)	C7—C12—C13—C14	−1.4 (4)
O1—C1—C2—N1	2.7 (3)	C12—C13—C14—C19	0.7 (4)
O2—C1—C2—C5	3.6 (4)	C12—C13—C14—C15	−178.8 (2)
O1—C1—C2—C5	−177.4 (2)	C13—C14—C15—C16	178.9 (2)
C2—N1—C3—C4	0.7 (4)	C19—C14—C15—C16	−0.6 (4)
Zn1—N1—C3—C4	−179.85 (18)	C14—C15—C16—C17	−0.7 (4)
C5—N2—C4—C3	−0.7 (4)	C15—C16—C17—C18	1.6 (4)
N1—C3—C4—N2	0.0 (4)	C16—C17—C18—C19	−1.2 (4)
C4—N2—C5—C2	0.7 (4)	C7—N3—C19—C18	176.9 (2)
C4—N2—C5—C6	178.2 (2)	C7—N3—C19—C14	−3.1 (4)
N1—C2—C5—N2	−0.1 (4)	C17—C18—C19—N3	179.8 (2)
C1—C2—C5—N2	179.9 (2)	C17—C18—C19—C14	−0.2 (4)
N1—C2—C5—C6	−177.4 (2)	C13—C14—C19—N3	1.6 (3)
C1—C2—C5—C6	2.6 (4)	C15—C14—C19—N3	−178.9 (2)
Zn1^iv^—O4—C6—O3	−171.95 (18)	C13—C14—C19—C18	−178.4 (2)
Zn1^iv^—O4—C6—C5	11.3 (4)	C15—C14—C19—C18	1.1 (3)

Symmetry codes: (i) −*x*+2, −*y*+2, −*z*+1; (ii) *x*, *y*+1, *z*; (iii) −*x*+2, −*y*+1, −*z*+1; (iv) *x*, *y*−1, *z*.

### Hydrogen-bond geometry (Å, °)

**Table:**

*D*—H···*A*	*D*—H	H···*A*	*D*···*A*	*D*—H···*A*
N3—H3N···O3	0.86 (2)	1.78 (2)	2.634 (3)	172 (3)
C13—H13···O2^v^	0.93	2.38	3.211 (4)	149
C16—H16···O3^vi^	0.93	2.49	3.377 (3)	159

Symmetry codes: (v) −*x*+1, −*y*+1, −*z*+1; (vi) *x*, −*y*+1/2, *z*+1/2.
